# Vestibular function following unilateral cochlear implantation for profound sensorineural hearing loss

**DOI:** 10.1186/s40463-016-0150-6

**Published:** 2016-06-14

**Authors:** Gavin J. le Nobel, Euna Hwang, Adrian Wu, Sharon Cushing, Vincent Y. Lin

**Affiliations:** Department of Otolaryngology Head and Neck Surgery, University of Toronto, 190 Elizabeth Street, Rm 3S438, RFE building, Toronto, ON M5G2N2 Canada; University of Toronto, Toronto, ON Canada; Sunnybrook Health Sciences Center, Toronto, ON Canada; Hospital for Sick Children, Toronto, ON Canada

**Keywords:** Cochlear implant, Vestibular dysfunction, Sensorineural hearing loss

## Abstract

**Background:**

Many Canadians are affected by sensorineural hearing loss (SNHL) and those with severe or profound hearing loss may have poor hearing function despite optimized hearing aids. Cochlear implants (CI) offer effective hearing rehabilitation for these patients, however, concern continues to exist regarding possible effects of CI on the vestibular system and balance.

The objective of this study was to conduct a pilot study assessing the effects of unilateral cochlear implantation (CI) on balance and the vestibular system in post-lingually deafened adults.

**Methods:**

Twelve patients were included in this pilot study and were assessed pre-operatively and at immediate, 1 week, and 1 month post-operative intervals. Assessments consisted of the dizziness handicap inventory (DHI), subjective visual vertical (SVV), and timed up-and-go testing (TUG). When applicable, testing was repeated with the CI on and off.

**Results:**

Many patients were found to have deviated SVV at pre-operative and post-operative assessments. However, statistically significant changes were not seen when comparing pre-operative and post-operative SVV or when comparing SVV with the CI on and with the CI off. DHI was found to improve in five patients and worsen in two patients, however, no statistically significant change was found in DHI scores or with TUG testing.

**Conclusions:**

This current pilot study does not indicate that CI surgery or implant activity influence vestibular or balance function, however, this pilot study is underpowered and greater numbers of patients would need be assessed to confirm these findings.

## Background

Many Canadians are affected by sensorineural hearing loss (SNHL), with approximately two thirds of individuals aged 75 or older affected. Those with severe or profound hearing loss may have poor hearing function despite optimized hearing aids [1]. Cochlear implants (CI) offer effective hearing rehabilitation for these patients, however, concern continues to exist regarding possible effects of CI on the vestibular system and balance. Vestibular dysfunction may affect a patient’s ability to form an accurate environmental percept and impact balance. Even in the absence of CI, the elderly are at significantly increased risk of balance dysfunction, carrying with it significant quality of life and economic burden [2]. Superimposed on this is the fact that patients with even mild hearing loss have an increased risk of falls and that this risk increases with more severe hearing loss [3]. Understanding balance in the hearing impaired adult population and the potential impact of CI on balance are key to improving outcomes in this population.

Owing to close anatomic and physiologic relationships with the cochlea [4–7], CI surgery and CI electrical activity have been associated with effects on the vestibular system. Histologic changes seen following CI surgery may result in impaired vestibular function. Moreover, specific vestibular function tests have suggested a corresponding decrement in vestibular function [4, 7, 8]. Further, electrical activity is known to influence vestibular structures and CI current can spread outside the cochlea and stimulate nearby neural structures [9, 10]. Vestibular function tests have been seen to change following CI and have been shown to be influenced by CI activity [4, 11]. We suggest these effects on the vestibular system may affect balance.

Thus far, balance tests have not demonstrated significant changes following CI [4, 6–8]. Many of the previous studies, however, have been limited by a retrospective design, lack of structured symptom assessment, small patient numbers, limited follow up, and variable surgical technique. As such, our current pilot study aims to demonstrate the feasibility of serially assessing patients for clinical manifestations of vestibular and balance changes following CI in a prospective cohort study. These testing protocols can then be implemented prospectively in larger patient groups to further assess for vestibular and balance changes that may occur in patients or subgroups of patients who undergo CI.

## Methods

Patients undergoing unilateral CI for SNHL as part of the Sunnybrook Cochlear Implant Program were recruited between March 2014 and Jan 2015. Ethics approval for this study was previously obtained from the Sunnybrook Research Ethics Board as part of an ongoing database involving clinical assessments of patient with CIs. Patients were excluded if they were unable to participate in all assessments or if they had undergone previous otologic surgery. Patient demographics and assessment data were recorded prospectively in a computer based database.

Demographics collected included the age and gender of the patient as well as the etiology of the hearing loss. Patients were assessed pre-operatively and post-operatively at immediate, 1 week, and 1 month evaluations by one of the authors. Patients symptoms were assessed with questionnaires and the Dizziness Handicap Inventory (DHI) was used pre-operatively and at 1 month post-operatively. Changes in DHI score were determined using minimal clinically important difference (MCID) of 4 points and a minimal detectable change (MDC) of 17 points [12, 13]. Patients were categorized as having mild, moderate, or severe DHI scores if they had scores between 0 and 30, 31 and 60, and greater than 60; respectively [14]. Clinical exam was was performed by one of the authors (an otolaryngology resident, a neurotology fellow, or neurotology staff physician) and consisted of subjective visual vertical (SVV), head-thrust (HT), and timed up-and-go (TUG) testing. HT testing consisted of horizontal canal testing and was not done with video recording. The recorded outcome for HT testing consisted of presence or absence of a corrective saccade. Previous studies have demonstrated substantial agreement between HT testing by different physicians [15, 16]. SVV testing was conducted in a sitting position with the axis of rotation paralell to the floor and was calculated as the average score of 10 tests. Test results were categorized as deviating towards the operated ear, neutral, or deviating away from the operated ear and an abnormal result was defined as greater than 2 degrees from vertical [17, 18]. Testing was done using the Clear Health Media iPod application (Subjective Visual Vertical; Clear Health Media Inc., Wonga Park, Vic, Australia). HT testing was conducted in a sitting position. TUG testing was measured as the elapsed time required for a patient to rise from a sitting position, walk ten feet and return, and then sit down. Testing was performed in a quiet clinic environment and, when applicable, testing was repeated with the implant device on and off.

Statistical analysis was conducted using SPSS statistical software (SPSS for Macintosh 22.0; SPSS Inc., Chicago, IL, US) with *p* < 0.05 considered statistically significant. Pre-operative SVV scores were compared with the post-operative SVV scores using the Friedman test. Effect size was determined using Kendall’s W test. This was used to, subsequently, determine the number of patients required an adequately powered study. SVV scores at 1 month with the CI off were compared with SVV scored with the CI on using the Wilcoxon signed-rank test. Pre-operative and 1 month DHI scores were compared using the Wilcoxon signed-rank test. Mean and median TUG scores were calculated. Pre-operative and post-operative TUG scores were compared using the paired *t*-test as were TUG scores with the CI on and the CI off.

## Results

**Fig. 1:**
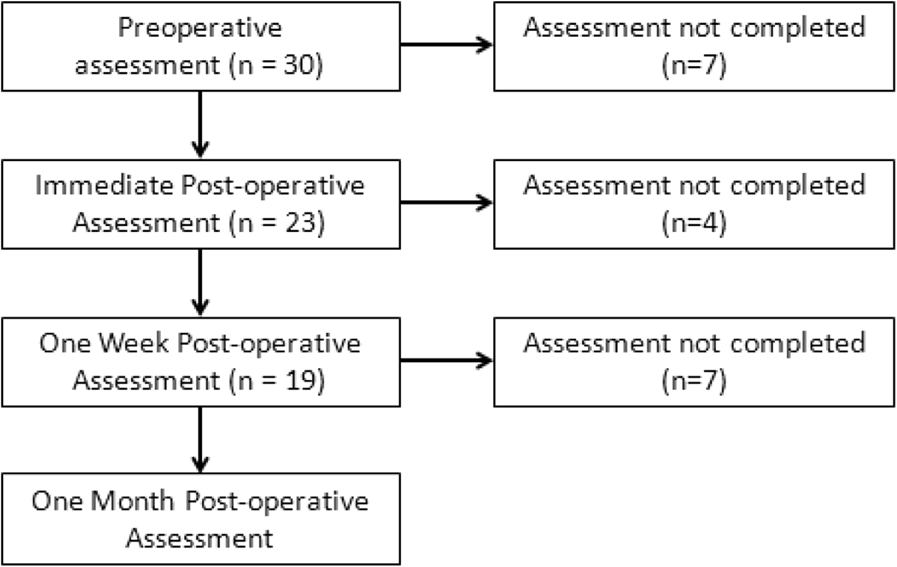
Patients enrolled in the study at each post-operative assessment and those excluded due to incomplete interval assessments

Thirty patients were initially enrolled, however, 18 of patients were excluded due to failure to complete all scheduled assessments. Figure 1 illustrates patients enrolled in the study at each interval assessment as well as which assessments patients did not complete and, consequently, when these patients were excluded from further study. This left 12 patients that completed evaluation at each of the pre-planned assessment intervals. Demographic of these patients as well as side of the implants and hearing loss etiologies are shown in Table 1.

**Table 1:** Patient demographics

age	20–78 (mean 56) years
gender	8 (67 %) male
implant side	9 (75 %) right
SNHL etiology	5 (41.6 %) SNHL not otherwise characterized
	1 (8.3 %) noise-induced
	1 (8.3 %) autoimmune
	1 (8.3 %) sudden SNHL
	1 (8.3 %) measles
	1 (8.3 %) ototoxic drug exposure
	1 (8.3 %) mitochondrial disease
	1 (8.3 %) congenital

**Fig. 2:**
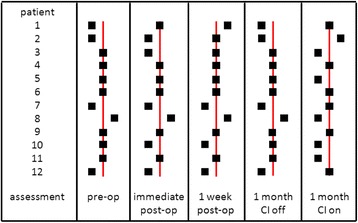
Subjective visual vertical for each patient at each assessment interval. Markers to the left of the neutral line indicate deviation away from the implant side. Markers to the right of the neutral line indicate deviation towards the implant side

Figure 2 demonstrates subjective visual vertical for each patient at each assessment interval. SVV testing was within normal limits for 58 % of pre-operative patients. In the immediate post-operative period, more patients exhibited abnormal SVV than at pre-operative assessment. SVV assessment 1 week post-operatively demonstrated that a majority of patients now had normal SVVs. When assessment was done at 1 month, at least 50 % of patients exhibited a normal SVV with the CI on or off. Interestingly, however, when an abnormal SVV was seen at 1 month, patients exclusively deviated away from their operated ear (Table 2). Post-operative SVV testing, however, did not demonstrate a statistically significant difference from pre-operative testing (*p* > 0.05). Kendall’s W test demonstrated an effect size of 0.065. With this effect size, approximately 500 patients would be required for an adequately power study using these assessment tools. SVV assessments with the CI off at 1 month, similarly, were not significantly different from SVV assessments with the CI on (*p* > 0.05).

**Table 2:** Pre-operative and post-operative SVV test results

	Deviation (relative to operated ear)		
	Away	Neutral	Towards
pre-operative	4	7	1
immediate post-op	5	5	2
1 week post-op	3	7	2
1 month post-op (CI Off)	5	7	0
1 month post-op (CI On)	6	6	0

**Fig. 3:**
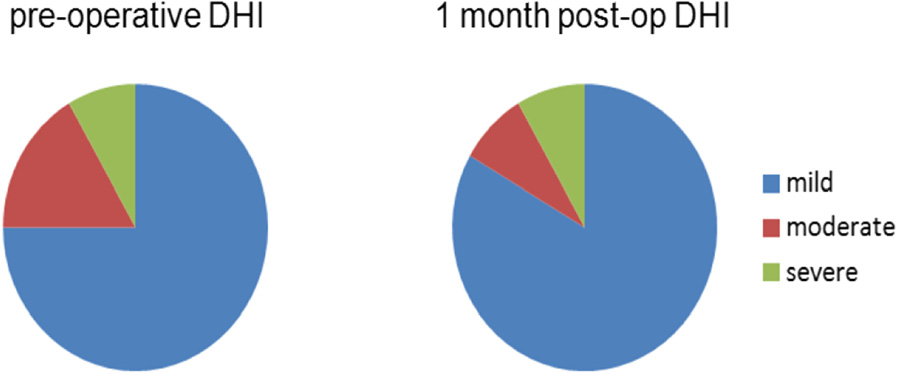
Pre-operative and 1 month post-operative DHI scores

The majority of patients exhibited mild DHI scores and no significant changes were seen in the proportion of patients with moderate & severe DHI scores (Fig. 3). Mean and median preoperative DHI scores were 16.5 and 4, respectively. A larger proportion of patients demonstrated improvement in DHI scores by MCID criteria, with 5 patients showing improved DHI scores and two patients showing worsened DHI scores. The same trend was seen when MDC criteria were applied, with three patients showing improvement and two patients showing worsened DHI scores. Mean and median DHI scores were 11 and 0 at 1 month follow up, respectively, and did not differ significantly from pre-operative scores (*Z* =−0.762, *p* = 0.446). No statistically significant differences were found when comparing pre-operative and post-operative TUG scores and the mean and standard deviation of TUG scores are shown in Table 3. Further, no statistically significant difference was found with TUG scores with the implant on and implant off (*t* =−0.701, *p* = 0.498).

**Table 3:** TUG scores and statistical analysis

	Timed up-and-go	Paired *t*-test	
	mean ± standard deviation (s)	t score	*p* value
pre-operative	12.8 ± 6.9		
1 week post-op	11.0 ± 4.1	1.65	0.13
1 month post-op (CI Off)	10.8 ± 4.3	2.04	0.07
1 month post-op (CI On)	10.6 ± 3.8	2.00	0.07

Head thrust testing did not demonstrate changes in the vestibular function in any of the operated ears between pre-operative assessments and 1 month follow up assessments with the CI on or off.

## Discussion

Adult patients undergoing unilateral CI for rehabilitation of SNHL were successfully enrolled in a prospective study assessing for clinical signs and symptoms of vestibular dysfunction following cochlear implantation. Twelve patients were enrolled and were able to complete assessments at each of the designated assessment intervals. Partial assessments of 18 other patients lead to their exclusion from the study.

Significant difficulty was encountered with enrolling patients in the study and ensuring that patients were assessed at every post-operative assessment, compromising the power of our study. This was largely a result of two issues. Firstly, a portion of patients declined to participate in preoperative or subsequent assessments, leading to exclusion from the study. In future studies, this would be challenging to improve upon. Secondly, pre-operative and post-operative assessments were schedule to coincide with patient visits mandated for other reasons. While it was felt that this approach would reduce inconvenience to the patients and potentially increase enrollment, study assessors were frequently unavailable during these times and this was the greatest contributor to limiting patient enrollment. This limitations could be overcome in future studies by having patients assessed at dedicated clinic assessments prior to their operation and by utilizing a study coordinator to ensure an assessor is available to perform follow up assessments for every patient. Since many more patients would be required for an adequately powered study, ensuring high patient recruitment and retention would be necessary.

The otolithic organs are thought to be the components of the vestibular system most likely to be affected by CI and this hypothesis is supported by histopathologic evidence as well as laboratory testing. Histopathologic changes are frequently found in the saccule and utricle but are less frequently found in the semicircular canals following CI [8]. These histopathologic changes may correlate with functional impairment of the otolithic organs. Further, studies of otolith function, including vestibular evoked myogenic potentials (VEMPs), have demonstrated impaired responses in patients following CI as compared with pre-operative testing [4, 19]. SVV testing operates on the principle that unilateral utricular hypofunction causes ocular torsion away from the side of the lesion and, consequently, deviation towards the side of the lesion [17]. Significant tilts of SVV have been seen following labyrinthectomy and vestibular neurectomy as well as with Meniere’s disease [17, 20]. As such, we hypothesized that SVV testing may be used to assess for any utricular dysfunction that may develop following CI.

Our study did not detect any statistically significant changes in SVV and, on this basis, utricular dysfunction does not appear to develop following CI. There are, however, several limitations which limit our study’s ability to detect utricular dysfunction following CI. Firstly, our study included too few patients to be adequately powered to detect statistically significant differences in the outcomes examined in this study. Performing an a-priori power calculation was not possible for this study as there was limited literature available to guide us in estimating mean SVVs or effect size. While the data gleaned from this study can be of assistance in providing an estimate of mean values and effect sizes for future studies, the validity of direct post-hoc power calculations is controversial [21]. Further, SVV testing may be an insensitive test for static utricular dysfunction and, thus, a static utricular lesion due to CI may go undetected [22]. Certainly, that decrement in otolithic functional tests, such as VEMPS, has been seen in other studies corroborates this hypothesis. Different methods of assessing otolithic dysfunction, such as VEMPs, may be more likely to detect static otolithic dysfunction. Lastly, many CI candidates have preexisting vestibular impairment due to shared etiology of cochleovestibular loss. Consequently, an insult to the vestibular system implied by CI may not cause an associated change in vestibular function and, thus, a change in SVV. Additional preoperative testing including VEMP testing would allow for subgroup analysis of those patients thought to be a greater risk of exhibiting SVV deviation.

In addition to potential changes associated with histopathologic changes in the vestibular system, CI electrical activity may potentially influence the vestibular system. Electrical activity is known to influence vestibular structures and Jin and colleagues have demonstrated changes in otolithic function associated with CI activity [10]. CI current has been seen to spread outside the cochlea and stimulate other nearby neural structures [9, 10]. The otolithic organs are the closest portions of the vestibular system to the cochlea and, it follows, the otolithic organs are most likely to be affected by CI electrical activity. In our study, no statistically significant difference was found when SVV testing was performed with the implant on and with the implant off at 1 month following surgery. It is possible that SVV may be insensitive in detecting the influence of CI electrical activity on otolithic organs. Further, our testing was conducted in a quiet clinic environment. It may be possible that if testing was conducted in a louder environment with resulting greater implant electrical activity, greater CI current spread could occur. This could potentially increase the likelihood of CI electrical activity influence on the vestibular system. This present study, however, suggests that CI electrical activity does not influence otolithic function.

Of note, it was found that patients with abnormal SVV at 1 month following CI exclusively demonstrated deviation away from the implanted ear. While this finding suggests a relative hyperfunction of the utricle in the implanted ear, we do not have a clear pathophysiologic mechanism to explain this. Given the limited number of patients included in this pilot study, it is possible that this represents a statistical anomaly, however, assessment of more patients could potentially shed light on this finding.

The majority of patients exhibited mild DHI scores preoperatively and post-operatively. Further, DHI scores were not found to change significantly between preoperative assessments and assessment at 1 month following surgery. DHI scores were found to change in a large proportion of patients by both MCID and MDC criteria. Surprisingly, however, a greater proportion of patients actually demonstrated improved DHI scores. This result is interesting, however, it may be confounded by some of the subcategories of the DHI such as the emotional subcategory that includes fear, frustration, avoidance behavior, and depression. It is possible that improved audition associated with the CI allows patients to regain confidence, thereby improving the patient’s perception of vestibular and balance disability.

Our study demonstrated no statistically significant changes in TUG scores when pre-operative scores were compared against post-operative scores. Further, no statistically significant difference was detected between TUG scores with the CI on and with the CI off. TUG testing was employed in this study due to low-cost and ease of implementation, however it is possibly insensitive to balance and mobility changes that could occur as a result of CI surgery or CI implant activity. Further testing including comprehensive tests of static and dynamic balance testing under more “real world” conditions could possibly elucidate more subtle balance changes that patients may experience due to CI.

## Conclusions

In this pilot, prospective cohort study, we were able to successfully enroll 12 patients undergoing unilateral CI for rehabilitation of profound SNHL. Although many patients exhibited abnormal DHI scores, SVV tests, and TUG testing, statistical analysis did not demonstrate significant differences between pre-operative and post-operative DHI, SVV, and TUG testing. Moreover, no statistically significant difference was found between SVV and TUG scores with the CI on or the CI off. Thus, with our current testing regimen, CI surgery and CI activity have not been seen to have deleterious effects on vestibular function and balance. However, this study is under powered to definitively assess whether changes in balance and vestibular function occur following CI and can be detected using these methods. This pilot study does, however, demonstrate the feasibility of enrolling patients undergoing cochlear implantation in a prospective trial to serially assess clinical manifestations of vestibular dysfunction.

## Abbreviations

CI, cochlear implant; DHI, dizziness handicap inventory; MCID, minimal clinically important difference; MDC, minimal detectable change; SNHL, sensorineural hearing loss; SVV, subjective visual vertical; TUG, timed up-and-go; VEMP, vestibular evoked myogenic potential
